# Comparison of Intense Pulsed Light Treatments including Upper Lid or Lateral Canthus in Patients of Meibomian Gland Dysfunction

**DOI:** 10.3390/jcm13123581

**Published:** 2024-06-19

**Authors:** Ji Sang Min, Ikhyun Jun, Tae-im Kim, Reiko Arita, Kyoung Yul Seo

**Affiliations:** 1 Institute of Vision Research, Department of Ophthalmology, Yonsei University College of Medicine, Seoul 03722, Republic of Korea; jsmansae@yuhs.ac (J.S.M.);; 2 Cornea Dystrophy Research Institute, Department of Ophthalmology, Yonsei University College of Medicine, Seoul 03722, Republic of Korea; 3 Itoh Clinic, Saitama 337-0042, Japan; 4 Lid and Meibomian Gland Working Group, Saitama 337-0042, Japan

**Keywords:** dry eye, intense pulsed light therapy, lateral canthus IPL treatment, meibomian gland dysfunction, upper lid IPL treatment

## Abstract

**Background:** To determine the differences in the effects of intense pulsed light (IPL) treatment when including the upper and lower lid or lateral canthus area in patients with meibomian gland dysfunction (MGD). **Methods:** Patients who underwent three IPL treatment sessions at 3-week intervals were divided into three groups according to the treatment sites: group A, lower lid; group B, upper and lower lids; and group C, lower lid and lateral canthal area. Before and after the IPL treatment sessions, we obtained the lid abnormality score (LAS), meibum expressibility (ME), meibum quality (MQ), lipid layer thickness (LLT), type I Schirmer test (ST), tear break-up time (TBUT) test, corneal fluorescein staining scores (CFSs), and Ocular Surface Disease Index (OSDI). **Results:** IPL treatment significantly improved LASs, ME, MQ, TBUT, CFS, and OSDI values in all groups. Differences in LAS values before and after IPL treatment were significantly greater in groups B and C than those in group A. **Conclusions:** IPL treatment encompassing the upper lid and lateral canthus together with the lower lid elicited additional improvement in patients with MGD. The additional effect on treating the lateral canthus was similar to the effect observed on the additional treatment of the upper lid.

## 1. Introduction

Meibomian gland dysfunction (MGD) causes evaporative dry eye (DE) because of a chronic abnormality of the meibomian gland, resulting in symptoms such as ocular surface burning and irritation, foreign body sensation, watering, fluctuating visual acuity, and red eye [1]. MGD may accompanied by specific lid margin signs such as irregular lid margin, vascular engorgement, plugged meibomian gland orifices, and anterior placement of the mucocutaneous junction [2,3], and these lid margin signs are used to diagnose MGD. There are also reports indicating that orifice plugging and lid margin thickening are correlated with the degree of MGD [4]. Additionally, a report suggested that lid margin thickness served as a strong indicator of age-related MGD [5].

Conventional treatment for MGD includes lid scrubbing, warm massage, topical corticosteroid and cyclosporine application, and oral antibiotics [6]. Despite the availability of various treatments, several patients with MGD fail to respond to therapy, leading to poor resolution or sustained symptoms.

Intense pulsed light (IPL) is a novel treatment with a wide application in MGD and is effective and safe [7,8,9,10,11,12]. Potential mechanisms of IPL in treating MGD include the destruction of blood vessel telangiectasia, liquefaction of meibum, downregulation of epithelial turnover, photomodulation, antimicrobiotic effects, and anti-inflammatory effects [13,14]. A recent study aimed to indirectly confirm that telangiectatic vessel ablation occurs through temperature changes in the eyelid skin after IPL treatment in patients with MGD [15].

When initially introducing IPL as a treatment for MGD, a protocol limiting the treatment site to the lower lid was proposed [6]. Subsequently, an IPL treatment protocol for treating the upper and lower lids together was introduced [16,17,18]. The effects of combined treatment of the lower and upper lids were found to be superior to the effects of treating the lower lid alone [19]. In addition, IPL treatment of the lower lid alone has been compared with combined treatment of the lower lid and lateral canthus treatment [20]. However, few studies have explored the differences in IPL treatment effects based on the treatment site in patients with MGD.

In this study, we examined the differences in IPL treatment effects when applied to the upper and lower lids or lateral canthal area in patients with MGD.

## 2. Materials and Methods

This study was conducted in accordance with the Declaration of Helsinki and was approved by the Institutional Review Board of Severance Hospital (No. 4-2023-0539). A retrospective analysis was performed using medical records of patients diagnosed with MGD who underwent three IPL treatment sessions at 3-week intervals between March 2021 and November 2022. The need for informed consent was waived owing to the retrospective nature of the study.

### 2.1. Patients

We included patients with a diagnosis of MGD based on the following criteria [21,22]: (1) At least one symptom, such as ocular fatigue, discharge, foreign body sensation, dryness, uncomfortable sensation, sticky sensation, pain, epiphora, itching, redness, heavy sensation, glare, excessive blinking, burning sensation, or ocular discomfort upon arising; (2) At least one abnormal lid margin finding, such as vascular engorgement, anterior or posterior replacement of the mucocutaneous junction, or irregular lid margin; (3) Plugged meibomian gland orifices and poor meibum expressibility (ME) in the target eye. IPL treatment was performed when there was insufficient treatment response to the conventional MGD therapy such as eyelid scrubbing, cyclosporin A eye drop, and oral antibiotics. Patients who met the following inclusion criteria were enrolled: (1) age > 18 years and (2) completion of three consecutive IPL treatments at 3-week intervals. We excluded patients with (1) missing DE and meibomian gland evaluation results before or after IPL treatment; (2) systemic diseases that may lead to DE disease; (3) a history of oral or topical retinoid use; (4) a history of intraocular surgery in the past 6 months; (5) a history of botulinum toxin or filler injection in the past month; (6) uncontrolled ocular disease; or (7) dark skin type, such as Fitzpatrick skin type V or VI [13].

### 2.2. Study Design

**Figure 1:**
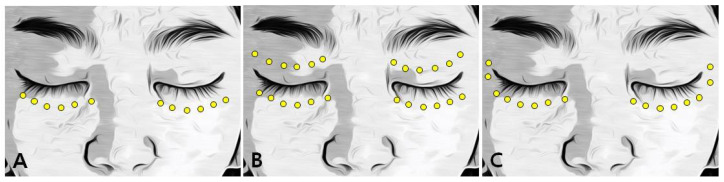
IPL treatment area. (**A**) Patients in group A received IPL treatment to only lower lid. (**B**) Patients in group B received IPL treatment to the upper and lower lid. (**C**) Patients in group C received IPL treatment to lower lid and lateral canthus.

Patients were categorized into the following three groups based on the IPL treatment site: group A, patients who received IPL treatment only to the lower eyelid; group B, those who received IPL treatment to the lower and upper eyelids; and group C, those who received IPL treatment to the lower eyelids and lateral canthus area (Figure 1). The ophthalmic examination results and the ocular surface disease index (OSDI) scores were compared before the first IPL treatment and after the third IPL treatment. The degree of treatment effect in each group was determined by differences in the ophthalmic examination results and OSDI scores before and after IPL treatment.

### 2.3. Clinical Assessment

For all patients, ophthalmic examination results and OSDI scores were evaluated before the first IPL treatment (3 weeks before IPL treatment) and after the third IPL treatment (3 weeks after IPL treatment). Ophthalmic examination included lipid layer thickness (LLT), tear break-up time (TBUT) test, corneal fluorescein staining (CFS) score, lid margin abnormalities score (LAS), ME, meibum quality (MQ), and type I Schirmer test (ST) result. To ensure independent testing results, the ophthalmic examination was conducted in the following order: (1) LLT; (2) CFS; (3) TBUT; (4) LAS; (5) ME; (6) MQ; (7) ST.

LLT was measured using a LipiView interferometer (TearScience, Morrisville, NC, USA). CFS, TBUT, LAS, ME, and MQ were assessed under slit-lamp examination. CFS scores and TBUT were measured by placing a single fluorescein strip (Haag-Streit International, Koniz, Switzerland), wetted with a drop of preservative-free normal saline, over the inferior tear meniscus. The tear break-up time (TBUT) assessed by stopwatch was measured after the patient blinked a few times. The average TBUT was then calculated from three repeated measurements. The pattern of corneal staining was evaluated according to the criteria of the Oxford Schema [22]. The lid margins and meibomian glands were microscopically examined after all other measurements were performed. The lid margin abnormalities were designated as 0 (absent) or 1 (present) for 4 criteria; lid margin irregularity, vessel engorgement, plugged meibomian glands, and anterior or posterior mucocutaneous junction displacement [23]. The score of each criteria was summed and designated as LAS, the severity in each criterion was not taken into consideration [23]. The degree of ME was determined by applying firm digital pressure over five glands of the lower lid and defined as follows: grade 0, all five glands were expressible; grade 1, three to four glands were expressible; grade 2, one to two glands were expressible; and grade 3, none of the glands were expressible. MQ was examined and assigned one of the following scores: grade 0, clear; grade 1, cloudy; grade 2, cloudy with granular debris; and grade 3, toothpaste-like. The scores for eight glands were summed to obtain a total score (maximum score: 24) [24]. The ST was performed without topical anesthesia by placing a standard paper strip (Eagle Vision, Memphis, TN, USA) on one-third of the mid-lateral portion of the lower fornix. The length of the wetting column was recorded in millimeters after 5 min.

The degree of treatment effects was evaluated by calculating the absolute value of the change in all ophthalmic exams and OSDI before and after IPL treatment.

### 2.4. IPL Procedure

After an ultrasonic gel was applied to the treated area, the eyes were protected using a Jaeger lid plate (Katena Products, Denville, NJ, USA). The M22 Optima device (Lumenis, Yokneam, Israel) was used to administer the IPL treatment. According to previous reports using the M22 machine for MGD treatment, the duration and interval of 6.0 ms and 60.0 ms had no serious side effects; therefore, the duration and interval were set the same as previous reports [15,17]. A 590 nm filter and a 6 mm cylindrical light guide were applied to the handpiece [13,15,25]. The fluence was set according to Fitzpatrick skin types (13–19 J/cm^2^), as described in previous studies [15,16,17]. A series of 6, 8, or 12 pulses were applied around the periocular area on the lower eyelids, upper eyelids, and the lateral canthus area (Figure 1). For a synergistic treatment effect, meibomian gland expression was performed using an Arita Meibomian Gland Compressor (Katena Products, Denville, NJ, USA) after the IPL treatment [13,15,17,25,26].

### 2.5. Statistical Analysis

To avoid double-organ bias, the data from the right eye were analyzed. Paired *t*-test was used to perform intergroup comparisons of ophthalmic examination results and OSDI scores before and after IPL treatment in each group. Furthermore, the degree of treatment effects in each group was assessed using one-way analysis of variance. Data were analyzed using SPSS 25.0 (IBM Corp., Armonk, NY, USA). Statistical significance was set at values of *p* < 0.05.

## 3. Results

The current study included 137 eyes of 137 patients diagnosed with MGD. Among them, 34 patients (34 eyes) who received IPL treatment solely on the lower lids were included in group A, 47 (47 eyes) who received IPL treatment on the lower and upper lids were included in group B, and 56 (56 eyes) who received IPL treatment on the lower lids and lateral canthal area were included in group C. The mean age of the patients was 53.31 ± 13.67 years, and 34 of them were men.

The LAS, ME, and MQ were significantly decreased after IPL treatment in all three groups. IPL treatment significantly reduced CFS scores and significantly enhanced TBUT in all three groups. OSDI scores were significantly decreased in all groups after IPL treatment (Table 1).

Table 2 present the differences in ophthalmic examination results and OSDI scores in all three groups before and after IPL treatment. There was a significant difference in LAS between groups A and B and between groups A and C. However, no differences were observed between groups B and C. Except for LAS, no significant differences were observed between the three groups in terms of changes in ophthalmic examination results and OSDI scores before and after IPL treatment. During and after IPL treatments, no serious adverse effects were associated with the treatment options.

## 4. Discussion

Numerous studies have shown that IPL treatment is effective in patients with MGD [8,9,11,14,17,18,19]. However, in most studies, IPL treatment was performed only on the lower lid [6,7,18,19] or both upper and lower lids [13,14,15,16,17,25]. Although the therapeutic effect of both treatment applications has been documented in patients with MGD, few studies have compared the differences in the therapeutic effects of IPL based on the treatment locations, such as the lower lid and upper lid. Therefore, comparing the effects of IPL treatment on the upper lid, lower lid, and lateral canthus in patients with MGD is beneficial in selecting the treatment modality.

 Several hypotheses have been suggested regarding the mechanism of IPL treatment in patients with MGD; however, the precise mechanism remains elusive [11,13]. A possible hypothesis is telangiectatic vessel ablation, and one study has attempted to confirm this mechanism [13]. The light energy from the IPL device is absorbed in the hemoglobin of the blood vessel, and then it generates heat for telangiectatic vessel ablation [11,12]. The eyelids are known to possess vascular supply in the form of anastomosis of the medial and lateral palpebral arteries [27]. The lateral palpebral artery located in the lateral canthus area branches to the lower and upper lids. The light-absorbed hemoglobin in the lateral canthus might move through the blood circulation system, such as the lateral palpebral artery, to the telangiectatic vessels of the upper lid tissue. Therefore, IPL treatment to the lateral canthus area is expected to elicit treatment effects on both the upper and lower lids.

According to the results of the current study, the effects of IPL treatment on the lower lid and lateral canthus area were equivalent to those on the upper and lower lids, and an additional treatment effect was observed when compared with IPL treatment limited to the lower lid. Due to retrospective study design, a decrease in telangiectasia could not be confirmed directly. Therefore, future research is required to directly confirm the reduction in telangiectasia in the upper and lower lid and lateral canthus area with IPL treatment.

Xue et al. [18] compared IPL treatment with four flashes on the lower lid only and IPL treatment with four flashes on the lower lid and an additional fifth flash on the lateral canthus. The authors reported that symptom improvement occurred more rapidly following treatment of the lower lid only when compared with combined treatment of the lower lid and lateral canthus. These effects were attributed to the fact that the transfer of thermal energy was easier, and the neuromodulatory effect occurred more effectively when IPL treatment of the lateral canthus was performed. Consistent with the findings of the previous report [18], the current study found that IPL treatment of the lateral canthus had an additional treatment effect on the parameters of MGD when compared with the IPL treatment limited to the lower lid. Xue et al. [18] suggested rapid improvements in symptom following IPL treatment of the lateral canthus; however, the authors only compared the tear film lipid layer grade and the meibomian gland capping grade between the cases where only the lower lid was treated and cases where both the lower lid and lateral canthus were treated, and no differences were reported in both groups. In the present study, the MGD and DE parameters were compared in two cases, revealing an additional treatment effect on the MGD parameter when IPL treatment was performed on the lateral canthus.

When initially introduced for MGD treatment, IPL treatment was applied to the lower lids only [6,8,9]. Subsequently, a method of treating the upper and lower lids was introduced [13,14,15,17,25], revealing that the combined treatment of the upper and lower lid elicited an additional treatment effect when compared with treating the lower lid alone [17]. IPL treatment performed on the upper lid has no serious side effects and is known to be safe [13,15,17,25,28]. However, the long-term safety of upper lid IPL treatment is yet to be evaluated. In clinical settings, upper-lid IPL treatment was found to be associated with greater patient discomfort, such as pain, than with lower-lid IPL treatment. According to the results of the current study, the effect of IPL treatment of the lower lid and lateral canthus area was comparable with that of IPL treatment of the upper and lower lids. Considering that the long-term safety of IPL treatment of the upper lid is yet to be established, IPL treatment of the lateral canthus can be pivotal.

The total energy of IPL treatment might be determined by the fluence, duration, area of the light guide, and the number of shots. In this study, the duration and area of the light guide are identical among groups; therefore, the differences in the total IPL treatment energy among groups are determined by the fluence and the number of shots. The fluence of the IPL treatment was determined by the patient’s Fitzpatrick skin types. Unfortunately, due to the retrospective study design, there was no information on the patients’ Fitzpatrick skin types. However, there were definite differences among the groups in the number of shots during IPL treatment. According to Table 2, there was no difference in treatment effect among the three groups in the remaining items other than LAS, and there was no difference in LAS between groups B and C. This indirectly indicates that there was no difference in IPL energy-corrected effect in this study. It is believed that a prospective and controlled study on the IPL energy-corrected effect will be needed in the future.

There are some limitations to our study. First, it used a retrospective design. Second, it was difficult to accurately compare the treatment effects because the total treatment areas of the two groups were not the same. Third, the duration of follow-up was limited to 3 weeks after the final treatment. Longer follow-up periods will be necessary to assess the long-term effectiveness and safety of IPL treatment in both light guides. Fourth, a decrease in telangiectasia was not directly confirmed in this study, and LAS is an insufficient metric for objective vascular ablation. Fifth, due to Meibomian gland expression followed after IPL treatment, there was a design deficiency because it does not allow observation of the true effect of IPL. Sixth, the total treatment area of IPL treatment was different among groups, so the outcome of IPL treatment of the lateral canthus may not be clearly shown. Seventh, LAS score was conducted under a relatively old scoring system [23] instead of a new scoring system [20] in this study. The effect of IPL treatment in the lateral canthus area under the new LAS scoring system might be required in the future.

In the current study, IPL treatment on the lower lid and lateral canthus showed an additional treatment effect when compared with the IPL treatment performed on the lower lid alone, and no difference was observed between the treatment effects of the upper and lower lids. A recent study revealed that combined treatment of the upper and lower lids is more effective than treatment of the lower lid alone [17] However, a greater proportion of patients experienced discomfort following IPL treatment of the upper lid than following IPL treatment of the lower lid. Therefore, IPL treatment of the lateral canthus, rather than treatment of the upper lid, could be considered as an alternative treatment strategy, eliciting the same effect with less pain.

## 5. Conclusions

Intense pulsed light treatment including the upper lid and lateral canthus together with the lower lid provided additional improvement in treating patients with MGD. The additional effect of the lateral canthus showed a similar effect with additional upper lid treatment.

## Figures and Tables

**Table 1:** Changes in the ophthalmic examinations and OSDIs after IPL treatment in each group.

	Group A			Group B			Group C		
	Before Treatment	After Treatment	*p*-Value	Before Treatment	After Treatment	*p*-Value	Before Treatment	After Treatment	*p*-Value
LAS	0.91 ± 0.64	0.47 ± 0.60	<0.001	1.96 ± 0.62	0.84 ± 0.68	<0.001	1.58 ± 1.00	0.44 ± 0.56	<0.001
ME	1.25 ± 0.55	0.50 ± 0.73	<0.001	1.67 ± 0.87	0.71 ± 0.73	<0.001	1.92 ± 0.87	0.83 ± 1.04	<0.001
MQ	19.17 ± 5.41	12.19 ± 6.55	<0.001	18.63 ± 5.81	10.16 ± 5.48	<0.001	22.07 ± 3.20	12.86 ± 6.47	<0.001
CFS	1.36 ± 0.96	0.41 ± 0.60	<0.001	1.16 ± 0.81	0.51 ± 0.79	<0.001	1.05 ± 0.88	0.25 ± 0.58	<0.001
TBUT (s)	3.16 ± 1.27	5.97 ± 2.10	<0.001	3.15 ± 1.38	5.52 ± 2.34	<0.001	2.71 ± 1.34	5.56 ± 5.97	<0.001
ST (mm)	11.39 ± 8.67	8.50 ± 5.21	0.085	12.97 ± 10.22	14.18 ± 10.02	0.235	13.98 ± 11.94	9.58 ± 7.33	0.064
LLT (ICU)	83.61 ± 19.76	73.11 ± 26.41	0.051	73.02 ± 25.51	69.20 ± 24.60	0.144	69.41 ± 27.61	61.75 ± 20.57	0.069
OSDI	40.16 ± 13.17	12.23 ± 9.16	<0.001	35.38 ± 19.97	15.37 ± 34.35	<0.001	42.37 ± 18.63	12.51 ± 11.00	<0.001

Abbreviations: group A, patients received IPL treatment to the lower and upper lid; group B, patients received IPL treatment to the lower lid and lateral canthus; group C, patients received IPL treatment to lower lid alone; IPL, intense pulsed light; ST, type 1 Schirmer test; TBUT, tear break-up time; CFS, corneal staining scores; LLT, lipid layer thicknesses; LAS, lid margin abnormalities score; ME, meibum expressibility; MQ, meibum quality, OSDI, Ocular Surface Disease Index. Data are shown as the mean ± standard deviation. Statistical analysis was performed using independent *t*-tests.

**Table 2:** Differences in ophthalmic examinations and OSDI values among lower and upper lid treatment, lower lid and lateral canthus treatment, and lower lid treatment.

	Group A (A)	Group B (B)	Group C (C)	*p*-Value		
				Difference between A and B	Difference between B and C	Difference between A and C
LAS	0.44 ± 0.50	1.11 ± 0.86	1.14 ± 0.92	<0.001	0.972	<0.001
ME	0.75 ± 0.50	1.03 ± 0.69	1.08 ± 0.93	0.088	0.899	0.135
MQ	6.97 ± 5.16	9.31 ± 6.04	9.42 ± 6.33	0.134	0.992	0.118
CFS	0.94 ± 0.92	0.84 ± 0.66	0.91 ± 0.74	0.975	0.861	0.776
TBUT (s)	2.80 ± 2.30	2.90 ± 2.11	3.05 ± 1.88	0.842	0.901	0.971
ST (mm)	2.89 ± 5.43	1.21 ± 5.11	4.40 ± 7.22	0.648	0.999	0.581
LLT (ICU)	10.50 ± 12.46	13.82 ± 18.56	7.66 ± 10.85	0.188	0.102	0.991
OSDI	27.92 ± 11.38	20.05 ± 11.56	30.06 ± 17.45	0.913	0.639	0.937

Abbreviations: group A, patients received IPL treatment to the lower and upper lid; group B, patients received IPL treatment to the lower lid and lateral canthus; group C, patients received IPL treatment to lower lid alone; IPL, intense pulsed light; ST, type 1 Schirmer test; TBUT, tear break-up time; CFS, corneal staining scores; LLT, lipid layer thicknesses; LAS, lid margin abnormalities score; ME, meibum expressibility; MQ, meibum quality, OSDI, ocular surface disease index. Data are shown as the mean ± standard deviation. Statistical analysis was performed using one-way ANOVA with Bonferroni adjustment.

## Data Availability

The datasets generated during and/or analyzed during the current study are not publicly available due to unavailability of ethical consent/approval for transfer of patient data to third parties, institutional mandate/guidelines but are available from the corresponding author on reasonable request.

## References

[1] Nelson J.D., Shimazaki J., Benitez-del-Castillo J.M., Craig J.P., McCulley J.P., Den S., Foulks G.N. (2011). The international workshop on meibomian gland dysfunction: Report of the definition and classification subcommittee. Investig. Ophthalmol. Vis. Sci..

[2] Raulin C., Greve B., Grema H. (2003). IPL technology: A review. Lasers Surg. Med..

[3] Geerling G., Tauber J., Baudouin C., Goto E., Matsumoto Y., O’Brien T., Rolando M., Tsubota K., Nichols K.K. (2011). The international workshop on meibomian gland dysfunction: Report of the subcommittee on management and treatment of meibomian gland dysfunction. Investig. Ophthalmol. Vis. Sci..

[4] Feng J., Wang J., Wu B., Shao Q., Zang Y., Cao K., Tian L., Jie Y. (2023). Association of meibomian gland morphology with orifice plugging and lid margin thickening in meibomian gland dysfunction patients. Int. Ophthalmol..

[5] Zhu H.Y., Liu X.Q., Yuan Y.Z., Wang D.H. (2022). Measurement of the Lid Margin Thickness in Meibomian Gland Dysfunction with Vernier Micrometer. Ophthalmol. Ther..

[6] Toyos R., McGill W., Briscoe D. (2015). Intense pulsed light treatment for dry eye disease due to meibomian gland dysfunction; a 3-year retrospective study. Photomed. Laser Surg..

[7] Craig J.P., Chen Y.H., Turnbull P.R. (2015). Prospective trial of intense pulsed light for the treatment of meibomian gland dysfunction. Investig. Ophthalmol. Vis. Sci..

[8] Vora G.K., Gupta P.K. (2015). Intense pulsed light therapy for the treatment of evaporative dry eye disease. Curr. Opin. Ophthalmol..

[9] Gupta P.K., Vora G.K., Matossian C., Kim M., Stinnett S. (2016). Outcomes of intense pulsed light therapy for treatment of evaporative dry eye disease. Can. J. Ophthalmol..

[10] Vegunta S., Patel D., Shen J.F. (2016). Combination Therapy of Intense Pulsed Light Therapy and Meibomian Gland Expression (IPL/MGX) Can Improve Dry Eye Symptoms and Meibomian Gland Function in Patients with Refractory Dry Eye: A Retrospective Analysis. Cornea.

[11] Giannaccare G., Taroni L., Senni C., Scorcia V. (2019). Intense Pulsed Light Therapy in the Treatment of Meibomian Gland Dysfunction: Current Perspectives. Clin. Optom..

[12] Tashbayev B., Yazdani M., Arita R., Fineide F., Utheim T.P. (2020). Intense pulsed light treatment in meibomian gland dysfunction: A concise review. Ocul. Surf..

[13] Yun J., Min J.S. (2022). Skin temperature change in patients with meibomian gland dysfunction following intense pulsed light treatment. Front. Med..

[14] Gao Y.F., Liu R.J., Li Y.X., Huang C., Liu Y.Y., Hu C.X., Qi H. (2019). Comparison of anti-inflammatory effects of intense pulsed light with tobramycin/dexamethasone plus warm compress on dry eye associated meibomian gland dysfunction. Int. J. Ophthalmol..

[15] Min J.S., Yoon S.H., Kim K.Y., Jun I., Kim E.K., Kim T.I., Seo K.Y. (2022). Treatment Effect and Pain during Treatment with Intense Pulsed-Light Therapy According to the Light Guide in Patients with Meibomian Gland Dysfunction. Cornea.

[16] Tang Y., Liu R., Tu P., Song W., Qiao J., Yan X., Rong B. (2021). A Retrospective Study of Treatment Outcomes and Prognostic Factors of Intense Pulsed Light Therapy Combined with Meibomian Gland Expression in Patients with Meibomian Gland Dysfunction. Eye Contact Lens.

[17] Chung H.S., Han Y.E., Lee H., Kim J.Y., Tchah H. (2023). Intense pulsed light treatment of the upper and lower eyelids in patients with moderate-to-severe meibomian gland dysfunction. Int. Ophthalmol..

[18] Xue A.L., Wang M.T.M., Ormonde S.E., Craig J.P. (2020). Randomised double-masked placebo-controlled trial of the cumulative treatment efficacy profile of intense pulsed light therapy for meibomian gland dysfunction. Ocul. Surf..

[19] Arita R., Itoh K., Maeda S., Maeda K., Furuta A., Fukuoka S., Tomidokoro A., Amano S. (2009). Proposed diagnostic criteria for obstructive meibomian gland dysfunction. Ophthalmology.

[20] Arita R., Minoura I., Morishige N., Shirakawa R., Fukuoka S., Asai K., Goto T., Imanaka T., Nakamura M. (2016). Development of Definitive and Reliable Grading Scales for Meibomian Gland Dysfunction. Am. J. Ophthalmol..

[21] Fitzpatrick T.B. (1988). The validity and practicality of sun-reactive skin types I through VI. Arch. Dermatol..

[22] Bron A.J., Evans V.E., Smith J.A. (2003). Grading of corneal and conjunctival staining in the context of other dry eye tests. Cornea.

[23] Arita R., Itoh K., Inoue K., Kuchiba A., Yamaguchi T., Amano S. (2009). Contact lens wear is associated with decrease of meibomian glands. Ophthalmology.

[24] Kim J.S., Lee H., Choi S., Kim E.K., Seo K.Y., Kim T.I. (2018). Assessment of the Tear Film Lipid Layer Thickness after Cataract Surgery. Semin. Ophthalmol..

[25] Kim M., Min J. (2022). Effect of Intense Pulsed-Light Treatment Using a Novel Dual-Band Filter in Patients with Meibomian Gland Dysfunction. J. Clin. Med..

[26] Chen Y., Li J., Wu Y., Lin X., Deng X., Yun E.Z. (2021). Comparative Evaluation in Intense Pulsed Light Therapy Combined with or without Meibomian Gland Expression for the Treatment of Meibomian Gland Dysfunction. Curr. Eye Res..

[27] Erdogmus S., Govsa F. (2007). The arterial anatomy of the eyelid: Importance for reconstructive and aesthetic surgery. J. Plast. Reconstr. Aesthet. Surg..

[28] Toyos R., Toyos M., Willcox J., Mulliniks H., Hoover J. (2019). Evaluation of the Safety and Efficacy of Intense Pulsed Light Treatment with Meibomian Gland Expression of the Upper Eyelids for Dry Eye Disease. Photobiomodul. Photomed. Laser Surg..

